# Analysis of 30 Putative *BRCA1* Splicing Mutations in Hereditary Breast and Ovarian Cancer Families Identifies Exonic Splice Site Mutations That Escape *In Silico* Prediction

**DOI:** 10.1371/journal.pone.0050800

**Published:** 2012-12-11

**Authors:** Barbara Wappenschmidt, Alexandra A. Becker, Jan Hauke, Ute Weber, Stefanie Engert, Juliane Köhler, Karin Kast, Norbert Arnold, Kerstin Rhiem, Eric Hahnen, Alfons Meindl, Rita K. Schmutzler

**Affiliations:** 1 Division of Molecular Gynaeco-Oncology, Department of Gynaecology and Obstetrics, University Hospital of Cologne, Cologne, Germany; 2 Center for Molecular Medicine Cologne, University of Cologne, Cologne, Germany; 3 Institute of Human Genetics, University of Cologne, Cologne, Germany; 4 Department of Gynaecology and Obstetrics, Klinikum rechts der Isar at the Technical University, Munich, Germany; 5 Department of Gynecology and Obstetrics, Technical University of Dresden, Dresden, Germany; 6 Division of Oncology, Department of Gynaecology and Obstetrics, University Hospital Schleswig-Holstein, Christian-Albrechts-University, Kiel, Germany; IFOM, Fondazione Istituto FIRC di Oncologia Molecolare, Italy

## Abstract

Screening for pathogenic mutations in breast and ovarian cancer genes such as *BRCA1/2*, *CHEK2* and *RAD51C* is common practice for individuals from high-risk families. However, test results may be ambiguous due to the presence of unclassified variants (UCV) in the concurrent absence of clearly cancer-predisposing mutations. Especially the presence of intronic or exonic variants within these genes that possibly affect proper pre-mRNA processing poses a challenge as their functional implications are not immediately apparent. Therefore, it appears necessary to characterize potential splicing UCV and to develop appropriate classification tools. We investigated 30 distinct *BRCA1* variants, both intronic and exonic, regarding their spliceogenic potential by commonly used *in silico* prediction algorithms (HSF, MaxEntScan) along with *in vitro* transcript analyses. A total of 25 variants were identified spliceogenic, either causing/enhancing exon skipping or activation of cryptic splice sites, or both. Except from a single intronic variant causing minor effects on *BRCA1* pre-mRNA processing in our analyses, 23 out of 24 intronic variants were correctly predicted by MaxEntScan, while HSF was less accurate in this cohort. Among the 6 exonic variants analyzed, 4 severely impair correct pre-mRNA processing, while the remaining two have partial effects. In contrast to the intronic alterations investigated, only half of the spliceogenic exonic variants were correctly predicted by HSF and/or MaxEntScan. These data support the idea that exonic splicing mutations are commonly disease-causing and concurrently prone to escape *in silico* prediction, hence necessitating experimental *in vitro* splicing analysis.

## Introduction

Between 1997 and 2012, more than 13.000 families fulfilling the criteria for hereditary breast and ovarian cancer were tested for mutations affecting the major susceptibility genes *BRCA1* and *BRCA2* [1], [2] by the German Consortium of Hereditary Breast and Ovarian Cancer (GC-HBOC). While pathogenic *BRCA1/2* mutations were detected in approximately 24% of the families (as of May 2012), a considerable amount of *BRCA1/2* variants were identified that are of unknown biological and clinical relevance, so called unclassified variants (UCV), including missense changes, small in-frame insertions or deletions, and potential splice site alterations. UCV are particularly problematic for cancer risk estimation and clinical management, as their functional implications are not immediately apparent [3]. Even though several splice site prediction algorithms are available, evaluation of UCV that possibly affect *BRCA1/2* pre-mRNA processing is challenging as it frequently requires experimental validation. Numerous *BRCA1/2* splicing mutations have been identified by using either mRNA derived from mutation carriers or by employing *BRCA1/2* minigene constructs [4], [5], [6], [7], [8], [9], [10]. The majority of these studies focuses on variants located within or in the close proximity of intronic splice sites only, suggesting that many mutations located deeper in the intron or exon that impair proper *BRCA1/2* pre-mRNA processing remain elusive.

Today, there is ample evidence that disease-causing splicing mutations are more prevalent than previously expected. An often-cited estimate of 15% reflects only mutations that are known to affect the splice sites [11]. When assayed directly for individual genes, up to 50% of disease-causing mutations are found to affect splicing and it has been proposed that even 60% of mutations that cause disease do so by disrupting splicing [12], [13]. This discrepancy is due to the finding that many human disease genes harbour exonic alterations that affect pre-mRNA splicing. Nonsense, missense and even translationally silent exonic mutations can impair gene activity by inducing the splicing machinery to skip the mutation-bearing exons. However, only a few exonic splicing mutations within *BRCA1* have been reported so far [5], [6], [14]. Based on these findings, experimental validation of putative *BRCA1/2* splicing mutations, both intronic and exonic, appears to be required. The pathogenic potential of putative splicing mutations is routinely estimated using *in silico* prediction analyses such as the maximum entropy model (MaxEntScan) [15] or the Human Splice Finder (HSF) algorithm [16]. In this study, we assessed the functional impact of 30 distinct *BRCA1* variants on pre-mRNA processing by employing bioinformatic prediction tools and experimental analysis of mRNA derived from carriers. Among the 24 intronic and 6 exonic variants analyzed, a total of 25 variants, including 4 missense mutations and 2 silent alterations were identified spliceogenic, either cause/enhance exon skipping or activation of cryptic splice sites, or both. Interestingly, 23 out of 24 intronic variants were correctly predicted by combined bioinformatic analyses, while 3 out of 6 exonic variants clearly escaped *in silico* detection. In summary, these data contribute to the recent knowledge of *BRCA1* splicing mutations and further highlight the importance of experimental splicing analysis particularly for exonic *BRCA1* variants and the need for improved bioinformatic prediction of exonic variants that affect the splicing machinery.

## Materials and Methods

### Probands and DNA isolation

Probands were recruited at the German consortium of hereditary breast and ovarian cancer (GC-HBOC) centres in Cologne, Dresden, Kiel or Munich. Genomic DNA was isolated from venous blood samples using the salting out method [17] or the QIAamp DNA Blood Maxi Kit (#51194, Qiagen, Hilden, Germany). Mutational screening was performed by denaturing high performance liquid chromatography (DHPLC) on all exons, followed by direct sequencing of conspicuous exons [18]. Ethical approval for this study was given by the institutional Ethics Committee of the University of Cologne, Germany (07-185, 10/18/2007). Written informed consent was obtained from all patients and control individuals.

### Reverse transcription PCR (RT-PCR)

Reverse transcription PCR (RT-PCR) was performed to determine effects of intronic and exonic sequence variants on *BRCA1* pre-mRNA processing. Total RNA was isolated from peripheral blood leukocytes using TRIzol Reagent (#15596-018, Invitrogen, Carlsbad, CA, USA). RNA concentrations were determined using a NanoDrop ND-1000 spectrophotometer (Peqlab, Erlangen, Germany). Reverse transcription was carried out by employing the Transcriptor High Fidelity cDNA Synthesis Kit (#05091284001, Roche Applied Science, Mannheim, Germany) using 500 ng of total RNA and oligo (dT)18 primers. Subsequent PCR were performed using the Qiagen Multiplex PCR Kit (#206145, Qiagen, Hilden, Germany), template-specific primers (table S2 A), and one microlitre of the RT reaction. PCR products were separated on 2.5% agarose gels and visualized by ethidium bromide staining. For long-range amplification of exon 11 and flanking sequences, we employed the Phusion Hot Start II High-Fidelity DNA Polymerase according to the manufacturer's protocol (# F-549S, Thermo Scientific, Bonn, Germany). PCR products were additionally analyzed by Sanger sequencing using ABI 3100 or ABI 3500xL Genetic Analyzers (Applied Biosystems, Carlsbad, CA, USA). When indicated, electrophoretically separated PCR products were purified from agarose gels using the QIAquick Gel Extraction Kit (#28704, Qiagen, Hilden, Germany). Densitometric analysis of band intensities was performed using the Quantity One software version 4.5.1 (BioRad, Munich, Germany).

### Quantitative RT-PCR

For real-time quantification of target gene expression, one-step real-time PCR was performed using the QuantiTect SYBR Green RT–PCR Kit (Qiagen, Hilden, Germany) on an Applied Biosystems StepOne Plus Real-Time PCR System (Applied Biosystems, Darmstadt, Germany). Each 20 µl RT–PCR mix contained 10 ng total RNA (4 ng/µl), 2 µl of the primer dilution, 10 µl Quanti-Tect SYBR Green RT-Master Mix and 0.2 µl QuantiTect RT Mix. One-step RT–PCR reactions were carried out in 96-well optical reaction plates, covered with Optical Adhesive Covers (Bioplastics, Landgraaf, Netherlands). Cycling conditions were as follows: 50°C for 30 min (reverse transcription step), 95°C for 15 min and 40 cycles of 94°C for 15 s, 60°C for 30 s and 72°C for 35 s. Real-time RT–PCR was conducted four times for each amplicon and each RNA sample. The comparative method of relative quantification (2^−ΔΔCt^) was used to calculate the relative expression levels of each amplicon. Results are given as mean ± SD. RT–PCR specificity of each PCR reaction was verified by melting curve analysis and confirmed by agarose gel electrophoresis. Amplicons have been designed to span exon borders to exclude false positive detection of genomic contaminations. Primers are listed in table S2 B.

### In silico analysis, databases and nomenclature

For splice site prediction, we employed the maximum entropy model (MaxEntScan) [15] and the Human Splice Finder (HSF) algorithm [16], which calculate splice junction strengths (MaxEntScan) or consensus values (CVs) (HSF), respectively, for the wild type and mutated sequences (http://www.umd.be/HSF/). For HSF, a ΔCV of 10% or more is considered significant based on empirical studies of known splicing mutations [16]. For MaxEntScan, a cutoff value of 20% has been suggested, though the cutoff is stated to be arbitrary [19]. In the provided tables the variants are described in both the traditional BIC nomenclature and the HGVS nomenclature based on the U14680.1 reference sequence for *BRCA1*. For comparison with the BIC website in the main text the description according to BIC is given. Genomic variation frequencies are given according to the 1000 Genomes (http://www.1000genomes.org), the Exome Variant Server (EVS; http://evs.gs.washington.edu/EVS/) the Breast Cancer Information Core (BIC; http://research.nhgri.nih.gov/bic/) databases and BRCA2006, the internal databases of the German Consortium of Hereditary Breast and Ovarian Cancer (GC-HBOC).

## Results

### BRCA1 mutations within invariant splice sites

**Figure 1:**
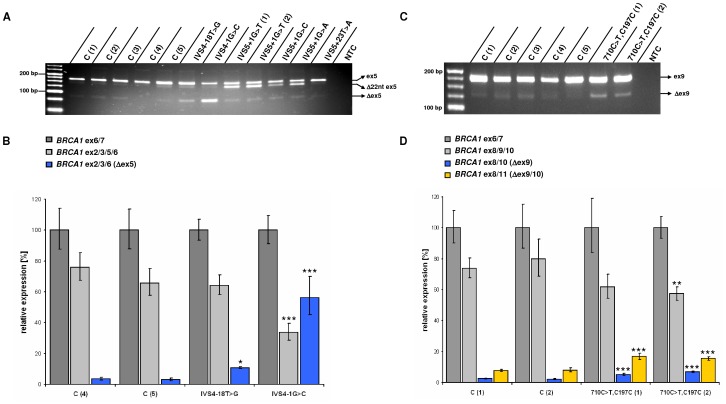
RT-PCR analyses of *BRCA1* exons 5 (A, B), 9 (C, D), and flanking sequences. ***A*** *)* Compared with controls C (1) to C (5), the variants *IVS4-18T>G* and *IVS4-1G>C* elevate exon 5 exclusion (Δex5), while *IVS5+1G>T*, *IVS5+1G>C* and *IVS5+1G>A* promote the usage of an upstream cryptic splice site, resulting in a 22 bp deletion on mRNA level (Δ22nt ex5). Regarding the variant *IVS5+1G>T*, two mRNA samples derived from two related mutation carriers were analyzed. NTC = no template control. Effects of the variant *IVS5+23T>A* on *BRCA1* pre-mRNA processing were not observed. ***B*** *)* Compared with two control samples, enhanced exon 5 skipping in *IVS4-18T>G* and *IVS4-1G>C* samples was confirmed by quantitative real-time RT-PCR analyses. Expression data are given as mean ± standard deviation (s.d.). Relative to an internal *BRCA1* control set to 100% (amplicon spanning exon 6 and 7 sequences, *BRCA1* ex6/7), the relative amounts of transcripts lacking exon 5 (*BRCA1* ex2/3/6) account for 3.49% (+1.01, −0.78) and 3.03% (+1.11, −0.81) in control samples, respectively, while the relative amounts of *BRCA1* ex2/3/6 transcripts are approximately 3fold increased in *IVS4-18T>G* samples (10.83%, +0.76, −0.71). *IVS4-1G>C* increases the relative amount of *BRCA1* ex2/3/6 transcripts to 56.21% (+13.77, −11.06), while the share of transcripts harbouring exon 5 sequences is significantly reduced. Three levels of statistical significance were discriminated: * = P<0.05, ** = P<0.01, *** = P<0.001 (t-test). ***C*** *)* The variant *710C>T,C197C* elevates skipping of exon 9 (Δex9) compared with controls. Total mRNA samples derived from two unrelated *710C>T, C197C* mutation carriers were analyzed. ***D*** *)* Enhanced exon 9 skipping was confirmed by quantitative real-time analysis. While transcripts lacking exon 9 (*BRCA1* ex8/10) account for 2.51% (+0.23, −0.21) and 2.14% (+0.35, −0.30) relative to the respective internal controls, the amounts of *BRCA1* ex8/10 mRNA species are approximately 2fold increased in samples derived from two independent patients carrying the *710C>T, C197C* variant (5.23%, +0.70, −0.62; 6.92%, +0.55, −0.51). Similar results were observed when analyzing the relative amounts of transcripts lacking exons 9 and 10 (*BRCA1* ex8/11). In controls, relative *BRCA1* ex8/11 levels account for 7.69% (+0.70, −0.64) and 8.04% (+1.30, −1.12) and 16.73% (+2.25, −1.98) and 15.48% (+1.23, −1.14) in samples derived from two independent *710C>T, C197C* carriers.

**Figure 2:**
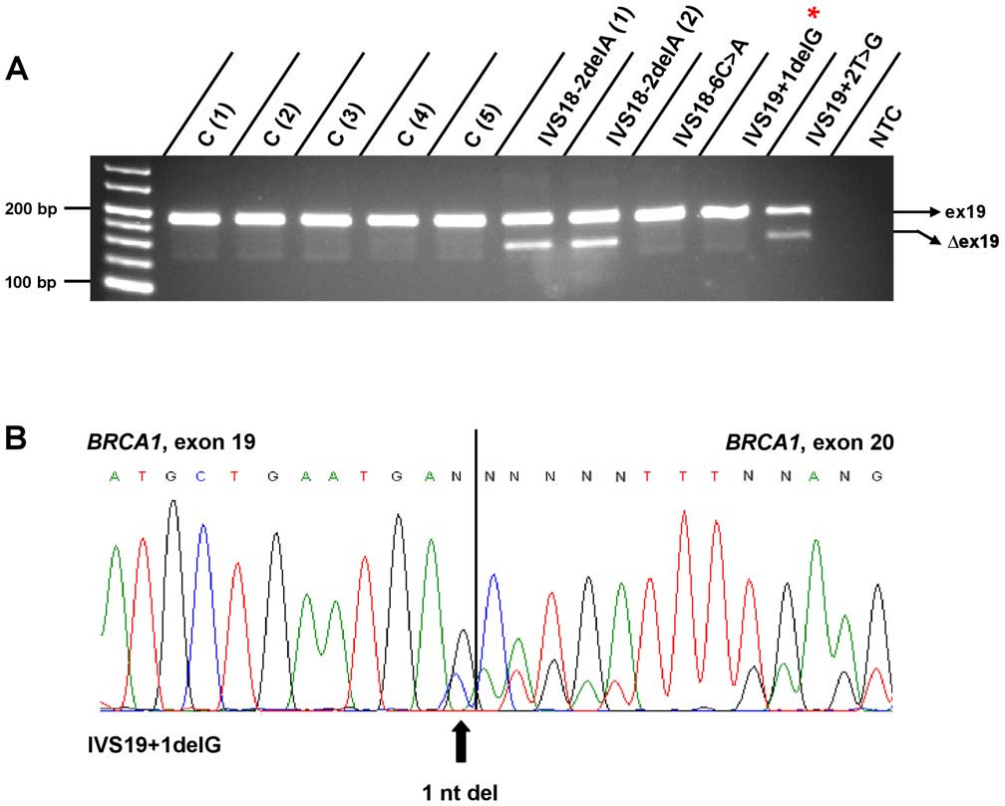
RT-PCR analyses of *BRCA1* exon 19 and flanking sequences. ***A*** *)* Compared with controls C (1) to C (5), the variants *IVS18-2delA* and *IVS19+2T>G* elevate exclusion of exon 19 (Δex19). Regarding the variant *IVS18-2delA*, two mRNA samples derived from two unrelated mutation carriers were analyzed. Effects of *IVS18-6C>A* on *BRCA1* pre-mRNA processing were not observed. *IVS19+1delG* did not associate with a suspicious splicing pattern as shown by RT-PCR followed by gel electrophoresis. * Note that *IVS19+1delG* causes a 1 nt deletion on transcript level not detectable by agarose gel electrophoresis. ***B*** *)* Direct sequencing of *IVS19+1delG* samples following RT-PCR revealed the deletion of the last nucleotide of exon 19 on mRNA level due to the activation of a cryptic splice site, which incorporates the last nucleotide of exon 19. NTC = no template control.

We analyzed a total of 12 *BRCA1* variants (derived from 14 independent cases) located within invariant donor or acceptor dinucleotides, all of which are predicted to be damaging according to both, HSF and MaxEntScan analyses (table S1). While some variants have previously been described on genomic level (6/12, see below), the assessment of their functional consequences for *BRCA1* pre-mRNA processing is pending in all cases. RT-PCR analyses paralleled by Sanger sequencing revealed all 12 variants to either cause aberrant exon exclusions or to activate nearby cryptic splice sites, or both. In detail, the vast majority of naturally occurring *BRCA1* transcripts carry exon 5, while some mRNA species either partially (*BRCA1*-Δ22ntex5) or completely lack exon 5 (*BRCA1*- Δex5) [20]. While these naturally occurring isoforms were detected in control samples, *IVS4-1G>C* markedly increases skipping of exon 5 (Figure 1 A, B). Three damaging mutations within the donor splice site of intron 5 (*IVS5+1G>T*, *IVS5+1G>A*, *IVS5+3A>G*) have been reported to enhance the usage of an upstream cryptic splice site, resulting in a 3′ 22 bp deletion of exon 5 on mRNA level (*BRCA1*-Δ22ntex5) [4], [20], [21], [22], [23]. In our cohort, we identified a *IVS5+1G>C* variant [24], which expectedly had similar effects (Figure 1 A). In contrast to these variants that quantitatively affect exon recognition, *IVS17-2A>G* (Figure S1 H), *IVS18+1G>C* [25] (Figure S1 H), *IVS18-2delA* [26] (Figure 2 A), *IVS19+2T>G* (Figure 2 A), *IVS21-1G>T* (Figure S1 K) and *IVS22+2delT* (Figure S1 K) cause the exclusion of the respective exons 18, 19, or 22, which was not observed in control samples. Another two variants, *IVS19-1G>T* and *IVS20-1G>A* [27], cause aberrant exon exclusions and, in addition, activate cryptic splice sites. *IVS19-1G>T* causes skipping of exon 20 and the generation of *BRCA1* mRNA species lacking the first 13 nt of exon 20 (Figure S1 I). *IVS20-1G>A* augments skipping of exon 21 and triggers the production of mRNA species lacking the first 8 nt of exon 21 (Figure S1 J). Besides these 10 variants described so far, the remaining 2 do not cause whole exon exclusions. *IVS2-1G>C* [28] promotes the activation of a cryptic splice site within exon 3, resulting in a mRNA isoform lacking the first 7 nt of exon 3 (Figure S1 A). *IVS19+1delG* did not associate with a suspicious splicing pattern as shown by gel electrophoresis of RT-PCR products. However, sequencing revealed the deletion of the last 3′ nucleotide of exon 19 on mRNA level due to the activation of a cryptic splice site, which includes the last nucleotide of that exon (Figure 2 A, B).

### Intronic BRCA1 variants outside invariant splice dinucleotides

In our cohort, we identified a total of 12 intronic variants located outside invariant splice sites, one of which has already been described on genomic level (*IVS16+3G>C*) and is considered damaging [29]. By employing the splice site prediction algorithms described above, *IVS16+3G>C* and five more variants (*IVS11+3A>G*, *IVS16+4A>G, IVS16+5G>A*, *IVS22+3A>T*, *IVS22+4A>G*) likely impair existing splice sites according to HSF and/or MaxEntScan, while the remaining variants appear to be neutral or below the respective thresholds (table S1). In line with prediction data, *IVS11+3A>G* compromises the existing intron 11 donor splice site, thus enhancing the abundance of the naturally occurring isoforms *BRCA1*- Δex11 and splice variants lacking 3309 nucleotides from exon 11 but retaining 117 nucleotides from the 5′ end of exon 11 (Figure S1 C) [30]. The variant *IVS16+6T>C* (Figure S1 E) has already been described to activate a cryptic intronic splice site resulting in the incorporation of 69 bases of the 5′ end of intron 16 at the junction of exons 16 and 17 [31], [32]. The nearby variants identified in our cohort (*IVS16+3G>C*, *IVS16+4A>G* and *IVS16+5G>A*) are predicted to impair the splice donor site of intron 16. As expected, retention of intronic sequences was also observed in each case (Figure S1 F). Sanger sequencing revealed the incorporation of 65 nt of the 5′end of intron 16 in all cases, including *IVS16+6T>C*. Both, *IVS22+3A>T* and *IVS22+4A>G*, cause the exclusion of exon 22 (Figure S1 L). A total of 5 out of 6 remaining variants, predicted as neutral or below the respective HSF and MaxEntScan thresholds, indeed do not affect *BRCA1* pre-mRNA processing in our analyses (*IVS5+23T>A*, *IVS9-34T>C*, *IVS18-6C>A*, *IVS20+15C>T*, *IVS21+13G>T*) (Figures 1 A; 2 A; S1 B, I, J). Even though predicted neutral, *IVS4-18T>G* appears to marginally compromise intron 5 acceptor splice site recognition, thereby increasing exon 5 skipping (Figure 1 A, B). Compared with controls, densitometric measurements of band intensities confirmed *IVS4-18T>G* to moderately elevate the abundance of *BRCA1*- Δex5 mRNA species relative to transcripts harbouring exon 5 (data not shown). To validate this finding, we performed quantitative real-time analyses to evaluate the effects of *IVS4-18T>G* on *BRCA1* exon 5 exclusion. While *BRCA1*- Δex5 represents a rare isoform in controls, the occurrence of *IVS4-18T>G* increases exon 5 exclusion reaching levels of significance compared with control samples (Figure 1 A, B).

### Exonic BRCA1 variants

Exonic alterations potentially affect splicing and thus, we analyzed the impact of 6 distinct exonic variants on *BRCA1* pre-mRNA processing (table S1). While 3 variants have previously been described on genomic level (see below), the functional consequences on *BRCA1* pre-mRNA splicing were unclear in all but one case (*710C>T,C197C*). All 6 variants locate in the close vicinity (≤3 nt) to the respective exon borders. Only 3/6 variants, *4304G>A,Q1395Q*, *4794G>A,E1559K* and *5193G>C,D1692H* [33] are predicted to be deleterious according to HSF and MaxEntScan algorithms (table S1). Concordantly, the silent mutation *4304G>A,Q1395Q*, affecting the last nucleotide of exon 12, causes exon 12 exclusion (Figure S1 D). *4794G>A,E1559K*, which is located at the last nucleotide of exon 15, activates a cryptic splice site resulting in the loss of the last 11 nt of exon 15 (Figure S1 E). 5193*G>C,D1692H*, which affects the last nucleotide of exon 17, activates a cryptic splice site in intron 17, causing the retention of 153 nucleotides of intron 17 within the spliced transcript. Additionally, 5193*G>C,D1692H* appears to enhance exon 17 skipping compared to controls (Figure S1 G). Noteworthy, *BRCA1* transcripts lacking exon 17 are also observed in controls and thus represent naturally occurring isoforms. *787A>G,K223R*, *527G>C,G1803A* [34] and 710C>T,C197C clearly escaped *in silico* analyses. *787A>G,K223R*, affecting the antepenultimate nucleotide of exon 10, causes exon 10 exclusion (Figure S1 B). *5527G>C,G1803A*, which affects the second nucleotide of exon 23, causes skipping of that exon (Figures S1 M). The remaining variant *710C>T,C197C* [35], [36], predicted as neutral, is located at the antepenultimate nucleotide of exon 9. Previous analyses demonstrated this variant to only slightly impair exon 9 recognition, which supports a nonpathogenic role for *BRCA1 710C>T,C197C* [37]. By RT-PCR analysis and Sanger sequencing, we confirm this variant to moderately enhance exon 9 skipping (Figure 1 C, D). Subsequent real-time PCR analysis revealed that *710C>T,C197C* increases the abundance of *BRCA1* transcripts lacking exon 9 and exons 9 and 10 about 2fold, reaching levels of significance compared with each control sample (Figure 1 C, D). Including *710C>T,C197C*, we in summary identified 6 exonic variants located in the close vicinity of the respective exon border to affect correct *BRCA1* pre-mRNA splicing (*787A>G,K223R*; *4304G>A,Q1395Q*; *4794G>A,E1559K*; *5193G>C,D1692H*; *5527G>C,G1803A*). For *787A>G,K223R*, direct sequencing of wild-type sized RT-PCR products following gel extraction revealed a heterozygous A/G signal at position 787, indicating that the *787A>G* transition impairs correct *BRCA1* pre-mRNA splicing in an incomplete manner and thus, mutant BRCA1 proteins carrying the K223R amino acid substitution may be expressed. In contrast, transcripts carrying the *4304G>A,Q1395Q*, *4794G>A,E1559K*; *5193G>C,D1692H* or *5527G>C,G1803A* variants were not detected.

## Discussion

Screening for pathogenic variants in breast and ovarian cancer genes *BRCA1/2*, *CHEK2* [38] and *RAD51C* [39] is common practice for individuals from high-risk families. However, test results may be ambiguous due to the presence of one or more unclassified variants (UCV) in the concurrent absence of clearly cancer-predisposing mutations. This scenario considerably hampers cancer risk estimation and clinical management. Therefore, it appears necessary to functionally characterize UCV and to develop appropriate UCV classification tools. A particular class of variants represent putative splicing alterations, which are frequently assessed by *in silico* prediction and functionally analyzed using either mRNA derived from mutation carriers or by employing minigene constructs [4], [5], [6], [7], [8], [9], [10]. While variants located in the canonical splice site dinucleotides that flank the exons are generally considered deleterious, more distant exonic and intronic variants require experimental characterization due to the risk of erroneous *in silico* prediction as demonstrated in this study. Moreover, when predicted deleterious, it frequently remains elusive whether deleterious variants cause exon skipping and/or activate cryptic splice sites which may be located distant to the wild-type sites, hence not covered by the prediction algorithms.

We investigated 30 rare *BRCA1* variants regarding their spliceogenic potential using *in silico* (HSF, MaxEntScan) along with *in vitro* mRNA analyses. Among those, all 12 variants located within the canonical splice sites were predicted damaging, which was in-line with our *in vitro* mRNA analyses. A total of 6 out of 12 intronic variants outside the canonical splice sites were predicted damaging by HSF and/or MaxEntScan, which was also confirmed. Among the 6 remaining intronic variants predicted neutral, we demonstrate *IVS4-18T>G* to marginally impair proper processing of *BRCA1* pre-mRNA as it causes an approximately three-fold increased abundance of *BRCA1* transcripts lacking exon 5. *IVS4-18T>G* is listed 4 times in patient databases (2× BIC, 2× BRCA2006) and was not found on control chromosomes according to the EVS database. Even though the rare variant *IVS4-18T>G* only moderately augments the relative amount of *BRCA1-* Δex5 mRNA species (Figure 1A, B), we can not exclude a potentially disease-modifying effect and thus consider its significance uncertain.

We hypothesized that exonic alterations that affect proper *BRCA1* pre-mRNA processing are more abundant than currently known and thus included 6 exonic variants in our study. Among those variants, all located in the close vicinity (≤3 nt) to the respective exon border, one silent and two missense alterations indeed were predicted damaging and cause substantial splice defects. The remaining variants (710C>T,C197C, 787A>G,K223R and 5527G>C,G1803A) were below the respective HSF and MaxEntScan thresholds. Interestingly, 787A>G,K223R as well as 710C>T,C197C and 5527G>C,G1803A clearly escaped *in silico* prediction. 5527G>C,G1803A, which affects the second nucleotide of exon 23, causes skipping of that exon (Figures S1 M) while *787A>G,K223R* impairs exon 10 recognition. Noteworthy, the latter variant disrupts *BRCA1* pre-mRNA processing in an incomplete manner and thus, BRCA1 proteins carrying the possibly damaging K223R amino acid substitution are likely to be translated (HumVar Score: 0.906, PolyPhen-2 prediction). The remaining, silent variant predicted neutral (710C>T,C197C) causes a two fold increased abundance of the naturally occurring, rare isoforms *BRCA1*-Δ9 and *BRCA1*-Δ9/10 compared to controls (Figure 1 C and D). This data is in accordance with the findings published by Dosil and co-workers, who previously have shown that 710C>T,C197C only marginally alters exon 9 recognition [37]. The splicing defect observed might be due to the fact that 710C>T,C197C, affecting the antepenultimate nucleotide of exon 9, creates a novel exonic splicing silencer motif (TATTGC/TAG) [37]. In case of a pathogenic effect, however, the frequencies of the 710C>T,C197C variant (rs1799965) are expected to be elevated in patient compared with control databases. According to the EVS database, the 710C>T transition is present on 12 out of 7020 control chromosomes indicating a carrier frequency of 0.34% (12/3510). 710C>T,C197C is listed 31 times in BIC (31/14866, carrier frequency of 0.21%) and 34 in the BRCA2006 databases (34/13287, carrier frequency of 0.26%). The carrier frequency data supposes a non-pathogenic role for the 710C>T,C197C variant which is in line with previous studies [23], [37], [40], while disease-modifying effects can not be excluded.

In summary, we investigated 30 unclassified *BRCA1* variants with putative effects on splicing, 25 of which were experimentally proven spliceogenic in peripheral blood leukocytes (PBL). The degree of likelihood of pathogenicity of each variant remains elusive and requires further investigation, including multifactorial likelihood analysis and other approaches [41]. While variants with severe impact on splicing (Table 1) may be considered as likely pathogenic (class 4) according to the classification system proposed by Plon and colleagues [42], variants with only partial effects on splicing such as IVS4-18T>G, 787A>G,K223R and 710C>T,C197C (Table 1) are particularly challenging and remain of uncertain clinical significance (class 3). With respect to the tissue-specific nature of pre-mRNA processing, splicing alterations caused by these variants in PBL might not fully reflect those in the tissues at risk. Regarding the value of *in silico* prediction algorithms used in this study, 23 out of 24 intronic variants were predicted correctly by combined *in silico* analysis (HSF, MaxEntScan). Noteworthy, the MaxEntScan prediction performance clearly exceeds that of HSF in our cohort. Besides *IVS4-18T>G*, the remaining 23 out of 24 intronic variants were properly predicted by MaxEntScan, while 4 intronic variants experimentally proven damaging (IVS11+3A>G, IVS16+3G>C, IVS22+3A>T, IVS22+4A>G, table S1) were below the HSF threshold [16]. This finding further highlights the value of using multiple *in silico* prediction algorithms to improve accuracy. Among the 6 exonic variants analyzed in our study, 4 *BRCA1* variants substantially disrupt proper pre-mRNA splicing, supporting the notion that exonic splicing mutations are more common than previously assumed [12], [13]. Interestingly, only a few exonic splicing mutations within *BRCA1* have been reported so far [5], [6], [14]. 3 out of 6 exonic variants proven spliceogenic escaped prediction, indicating that *in silico* analysis currently performs relatively poor for exonic alterations [14], which highlights the need for improved bioinformatic prediction tools. Given the fact that prediction of ESE and ESS is also not yet fully accurate [43], [44], [45], [46], *in vitro* splicing analysis of exonic variants located close to the respective exon border is required and might be performed on a routinely basis.

**Table 1:** Classification of putative *BRCA1* splicing mutations.

BIC nomenclature	HGVS nomenclature	*in vitro* splicing result	*in vitro* splicing result (HGVS)	protein change (HGVS)	Family ID	Proband(s) analysed (phenoptype,onset)	Family history	Ethnicity
**A: Severe impact on splicing** *								
***BRCA1*** ** variants within invariant splice sites**								
IVS2−1G>C	c.81−1G>C	Δ7nt 5′ of exon 3	r.81_87del	p.Leu30*	09_2506	#001 (n.a.)	BC (40 y), 4× OC (40 y, 52 y, 65 y, 68 y)	European
IVS4−1G>C	c.135−1G>C	enhanced Δ exon 5	enhanced r.135__212del	p.[Phe45_Lys71del]	09_3489	#001 (n.a.)	4× BC (30 y, 33 y, 41 y, 47 y), 2× OC (50 y, 70 y*)	Afghan
IVS5+1G>C	c.212+1G>C	enhanced Δ22nt 3′ of exon 5	enhanced r.191_212del	p.Cys64*	09_1855	#001 (BC, 38 y)	1× BCbil (33 y+33 y), 3× BC (35 y, 38 y*, 42 y), 1× OC (55 y*)	European
IVS17−2A>G	c.5075−2A>G	Δ exon 18	r.5075_5152del	p.Asp1692_Trp1718delinsGly	03_0847	#001 (BC+OC, 42 y+47 y)	1× BC+OC (42 y+47 y*)	European
IVS18+1G>C	c.5152+1G>C	Δ exon 18	r.5075_5152del	p.Asp1692_Trp1718delinsGly	09_0879	#002 (BC, 35 y)	3× BC (35 y*, 71 y, 73 y)	European
IVS18+1G>C	c.5152+1G>C	Δ exon 18	r.5075_5152del	p.Asp1692_Trp1718delinsGly	09_0756	#002 (BC, 72 y, male)	3× BC (37 y, 50 y, 72 y*)	European
IVS18−2delA	c.5153−2delA	Δ exon 19	r.5153_5193del	p.Trp1718Serfs*1	09_0329	#001 (n.a., MTX)	6× BC (27 y*, 31 y*, 35 y*, 48 y, 50 y, 60 y)	European
IVS18−2delA	c.5153−2delA	Δ exon 19	r.5153_5193del	p.Trp1718Serfs*1	09_1131	#001 (n.a.)	1× BCbil (43 y+55 y*) together with OC (67 y*)	European
IVS19+1delG	c.5193+1delG	Δ last nt of exon 19	r.5193del	p.Glu1731Aspfs*33	09_2891	#001 (BC, 32 y)	1× BC (32 y*), 1OC (70 y*)	European
IVS19+2T>G	c.5193+2T>G	Δ exon 19	r.5153_5193del	p.Trp1718Serfs*1	09_2062	#001 (BC, 29 y)	2× BC (29 y*, 43 y)	European
IVS19−1G>T	c.5194−1G>T	Δ exon 20, Δ13nt 5′ of exon 20	r.[5194_5277del, 5194_5206del]	p.[His1732_Lys1759del, His1732Serfs*28]	09_1932	#001 (BCbil, 37 y+40 y)	1× BCbil (37 y+40 y*), 2× BC (43 y, 71 y)	European
IVS20−1G>A	c.5278−1G>A	enhanced Δ exon 21, Δ8nt 5′ of exon 21	enhanced r.5278_5332del, r.5278_5286del	p.[Phe1761Asnfs*13, Phe1761Glyfs*66]	09_3614	#001 (BC, 41 y)	2× BC (41 y*, 42 y)	European
IVS21−1G>T	c.5333−1G>T	Δ exon 22	r.5333_5406del	p.Asp1778Glyfs*26	09_1499	#001 (n.a.), #002 (BC, 37 y), #003 (BCbil, 39 y+56 y)	2× BCbil (39 y+56 y*, 56 y+56 y), 2× BC (37 y*, 65 y)	European
IVS22+2delT	c.5406+2delT	Δ exon 22	r.5333_5406del	p.Asp1778Glyfs*26	09_1288	#001 (BCbil, 38 y+39 y), #002 (BCbil, 29 y+43 y), #006 (BronC, 85 y)	2× BCbil (38 y+39 y*, 29 y+43 y*)	European
**Intronic ** ***BRCA1*** ** variants outside invariant splice sites**								
IVS11+3A>G	c.4096+3 A>G	enhanced Δ exon 11, Δ3309nt 3′ of exon 11	enhanced r.[671_4096del, 787_4096del]	p.[Ala224_Leu1365del, Ser264_Leu1365del]	12_0909	#001 (BC, 62 y)	3× BC (62 y*, 81 y, 51 y), 1× OC (55 y)	European
IVS16+3G>C	c.4986+3 G>C	Ins 65 nt intron 16	r.4986+1_4986+65ins	p.Met1663Valfs*14	09_0351	#001 (BC, 33 y)	2× BC (32 y, 33 y*), 1× BC+OC (39 y, 64 y), 1OC (70 y)	European
IVS16+4A>G	c.4986+4 A>G	Ins 65 nt intron 16	r.4986+1_4986+65ins	p.Met1663Valfs*14	12_0899	#001 (BCbil, 34 y+50 y), #009 (BC, 31 y)	1× BCbil (34 y+50 y*), 2× BC (31 y*, 40 y)	European
IVS16+5G>A	c.4986+5 G>A	Ins 65 nt intron 16	r.4986+1_4986+65ins	p.Met1663Valfs*14	09_4089	#001 (BC, 36 y)	4× BC (20 y, 36 y*, 50 y, 50 y)	Arabian
IVS22+3A>T	c.5406+3 A>T	Δ exon 22	r.5333_5406del	p.Asp1778Glyfs*26	TU367	#20226 (BC, 40 y)	2× BC (40 y*, 52 y)	European
IVS22+4A>G	c.5406+4 A>G	Δ exon 22	r.5333_5406del	p.Asp1778Glyfs*26	GH188	#11896 (OvCa, 60J)	1× OV (60 y*), DCIS (64 y), OvX (38 y*)	European
**Exonic ** ***BRCA1*** ** variants**								
4304G>A,Q1395Q	c.4185G>A, p.Gln1395Gln	Δ exon 12	r.4097_4185del	p.Gly1366Alafs*7	TU235	#19896 (OC, 61 y)	4× BC (34 y*, 43 y, 68 y, 57 y), 2× OC (60 y, 61 y*)	European
4794G>A,E1559K	c.4675G>A, p.Glu1559Lys	Δ11nt 3′of exon 15	r.4665_4675del	p.Gln1556Glyfs*13	09_3575	#003 (OC, 50 y)	1× BC (44), 2× OC (47 y, 50 y*)	European
5193G>C,D1692H	c. 5074G>C, p.Asp1629His	Ins 153 nt intron 17, enhanced Δ exon 17	r.5074+1_5074+153ins, enhanced 4987_5074del]	p.[Asp1692Gly*fs* *14, Val1665Serfs*7]	09_3943	#001 (BC, 27 y)	1× BC (27 y*)	European
5527G>C,G1803A	c.5408G>C, p.Gly1803Ala	Δ exon 23	r.5407_5467del	p.Gly1803Glnfs*10	09_ 2219	#001 (OC, 45 y)	1× BC (41 y), 4× OC (45 y*, 45 y, 45 y*, 53 y)	European
**B: Partial impact on splicing** *								
IVS4−18T>G	c.135−18T>G	enhanced Δ exon 5	enhanced r.135__212del	p.[Phe45_Lys71del]	09_1411	#001 (BC, 45 y)	3× BC (45 y*, 45 y, 83 y)	European
710C>T,C197C	c.591C>T, p.Cys197Cys	enhanced Δ exon 9 and 9/10	enhanced r.548_593del, r.548_670del	p.[Gly183Cysfs*16, Gly183_Lys223del]	09_1472	#001 (n.a.)	2× BC (36 y, 52 y)	European
710C>T,C197C	c.591C>T, p.Cys197Cys	enhanced Δ exon 9 and 9/10	enhanced r.548_593del, r.548_670del	p.[Gly183Cysfs*16, Gly183_Lys223del]	09_2106	#001 (BCbil, 50 y+55 y)	2× BCbil (50 y+55 y*, 59 y+65 y), 4× BC (36 y, 65 y, 70 y, 70 y)	European
787A>G,K223R	c.668A>G, p.Lys223Arg	enhanced Δ exon 10	enhanced r.594_670del	p.Val199Cysfs*2, p.Lys223Arg	12_0621	#001 (BC, 32 y), #002 (BC, 58 y)	4× BC (32 y*, 52 y, 58 y*, 40 y);	European
**C: No effect on splicing observed** *								
IVS5+23T>A	c.212+23 T>A	/	**/**	/	09_3716	#001 (BC, 40 y)	2× BC (40 y*, 50 y)	European
IVS9−34T>C	c.594−34T>C	/	/	/	09_2602	#001 (BC, 21 y)	2× BC (21 y*, 44 y)	Turkish
IVS18−6C>A	c.5152−6C>A	/	/	/	09_1469	#001 (DCIS, 53 y)	2× BC (53 y*, 75 y), 1× DCIS (53 y*)	European
IVS20+15C>T	c.5277+15C>T	/	/	/	09_2627	#001 (BC, 35 y)	1× BC (35 y*)	European
IVS21+13G>T	c.5332G>T	/	/	/	09_2376	#001 (BC, 42 y)	2× BC (42 y*, 45 y)	European

**A:** Variants that severely affect splicing, **B:** Variants having a partial effect only, and **C:** Variants that do not affect processing of *BRCA1* pre-mRNA species in PBL.

* Variants with severe impact on splicing are considered as likely pathogenic (class 4) according to the classification system proposed by Plon et al., [42], while variants with only partial effects on splicing remain of uncertain clinical significance (class 3). Variant descriptions (BIC nomenclature, HGVS nomenclature), consequences on transcript- and protein levels, family IDs, analyzed index patients (phenotypes, age at onset), family histories (phenotypes, age at onset) and ethnic backgrounds are given. Family members carrying the same *BRCA1* variant are indicated (asterisk). All other listed family members were not available for analysis. Abbreviation: BC = breast cancer; OC = ovarian cancer; n.a. = not affected; bil = bilateral; ProC = prostate carcinoma; MTX = mastectomy; DCIS = Ductal carcinoma *in situ*.

## Supporting Information

Figure S1 RT-PCR analyses of *BRCA1* exons 3 (A), 10 (B), 11 (C), 12 (D), 15 (E), 16 (F), 17 (G), 18 (H), 20 (I), 21 (J), 22 (K, L) and 23 (M). The topmost band in lane *IVS2-1G>C* (A) and the middle bands in lanes *IVS9-2A>C* (B), *IVS21-1G>T* (K) and *IVS22+2delT* (K) could not be identified as additional *BRCA1* isoforms by direct sequencing and thus appear to be unspecific (data not shown). RT-PCR signals suggested to be unspecific are marked with red asterisks. In case of the variants *IVS16+4A>G* (F), *IVS18+1G>C* (H), *IVS21-1G>T* (K) and *IVS22+2delT* (K), mRNA samples derived from two or three mutation carriers were analyzed, which are unrelated in case of *IVS18+1G>C*. The variant *IVS9-2A>C* (B) [32], [47], which causes exon 10 skipping [48], was used as positive control. *IVS20-14C>G* (J), classified as neutral [49], was used as a negative control.
(PPT)

Table S1 **Classification, frequencies and** ***in silico*** **characterization of analyzed variants.** ***A:*** *BRCA1* mutations within invariant splice sites; ***B:*** Intronic *BRCA1* variants outside invariant splice sites; ***C:*** Exonic *BRCA1* variants*;* BIC, EVS and 1000 Genomes entries are as of 02/23/2012. BRCA2006 data of the GC-HBOC are as of 04/10/2012. EVS data refers to variation frequencies in the European/American population (rs numbers are given only when frequency data is available). Valuation of variants by the BIC steering committee is given in brackets, when available (yes = clinically important). The consensus values (CVs) for wildtype and mutant splice sites provided by HSF analysis are shown. For HSF prediction, a ΔCV of 10% or more is considered significant. For MaxEntScan analysis, a cutoff value of 20% has been suggested. Differences considered significant are shown in bold./ = no difference between for wildtype and mutant splice sites according to HSF or MaxEntScan.
(DOC)

Table S2 Oligonucleotides used for non-quantitative RT-PCR (A) and quantitative real-time RT-PCR analyses (B).
(DOC)
