# Supplementary material for: Analysis of 30 Putative BRCA1 Splicing Mutations in Hereditary Breast and Ovarian Cancer Families Identifies Exonic Splice Site Mutations That Escape In Silico Prediction
Source: PLoS One. 2012 Dec 11;7(12):e50800. doi: 10.1371/journal.pone.0050800 (PMC3519833; doi:10.1371/journal.pone.0050800)
Supplement: Figure S1 — RT-PCR analyses of BRCA1 exons 3 (A), 10 (B), 11 (C), 12 (D), 15 (E), 16 (F), 17 (G), 18 (H), 20 (I), 21 (J), 22 (K, L) and 23 (M). The topmost band in lane IVS2-1G>C (A) and the middle bands in lanes IVS9-2A>C (B), IVS21-1G>T (K) and IVS22+2delT (K) could not be identified as additional BRCA1 isoforms by direct sequencing and thus appear to be unspecific (data not shown). RT-PCR signals suggested to be unspecific are marked with red asterisks. In case of the variants IVS16+4A>G (F), IVS18+1G>C (H), IVS21-1G>T (K) and IVS22+2delT (K), mRNA samples derived from two or three mutation carriers were analyzed, which are unrelated in case of IVS18+1G>C. The variant IVS9-2A>C (B) [32], [47], which causes exon 10 skipping [48], was used as positive control. IVS20-14C>G (J), classified as neutral [49], was used as a negative control. (PPT) [file pone.0050800.s001.ppt]

## Slide 1
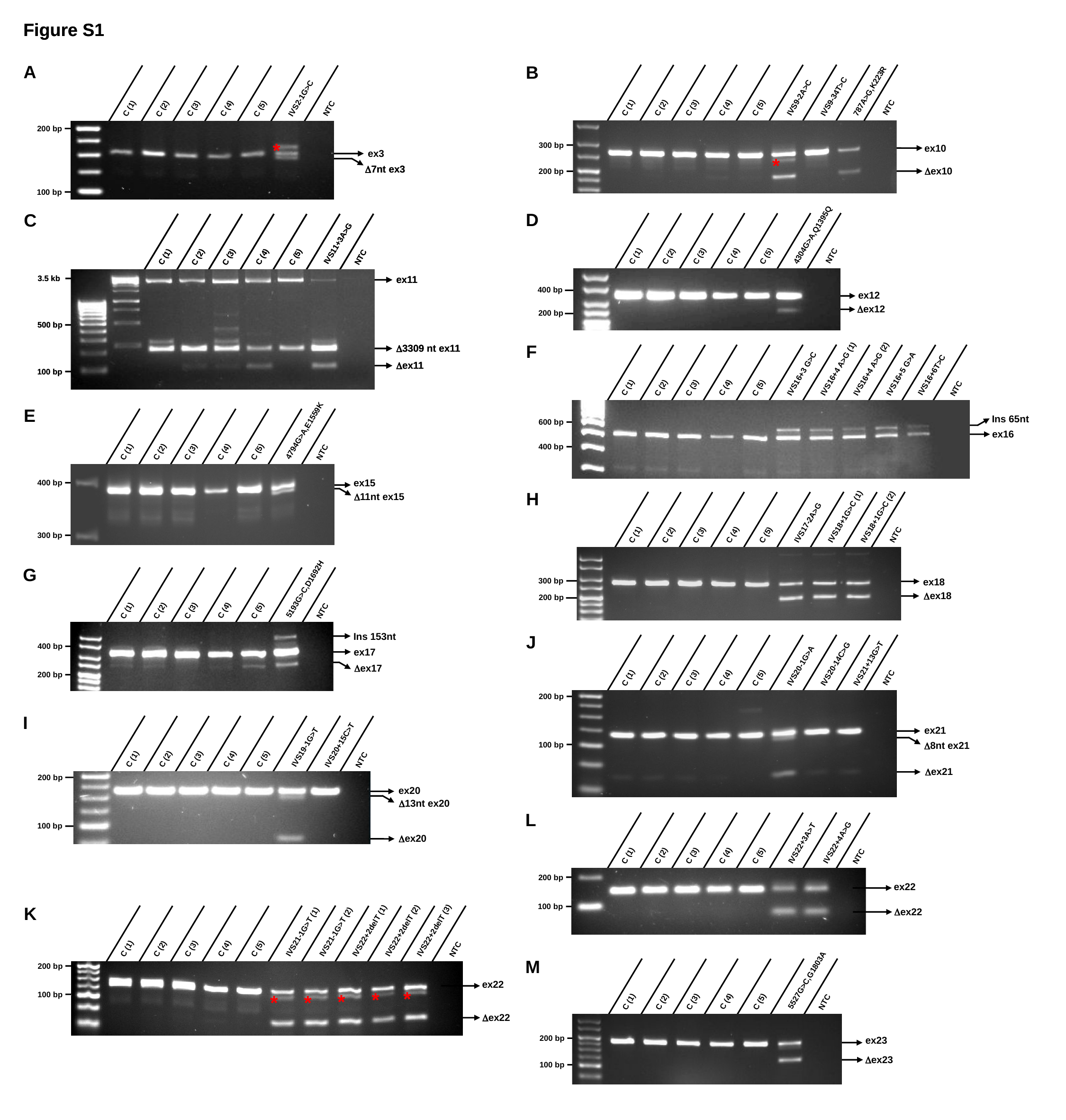

Figure S1
Figure S1
C (1)
C (3)
IVS2-1G>C
NTC
C (4)
C (5)
C (2)
200 bp
*
ex3
100 bp
787A>G,K223R
IVS9-2A>C
IVS9-34T>C
C (1)
NTC
C (5)
C (2)
C (3)
C (4)
300 bp
ex10
Δex10
200 bp
*
A
B
Δ7nt ex3
Δ7nt ex3
4304G>A,Q1395Q
C (1)
NTC
C (5)
C (3)
C (2)
C (4)
400 bp
ex12
Δex12
200 bp
IVS11+3A>G
IVS11+3A>G
C (1)
C (1)
NTC
NTC
C (5)
C (5)
C (3)
C (3)
C (2)
C (2)
C (4)
C (4)
3.5 kb
3.5 kb
ex11
ex11
500 bp
500 bp
Δ3309 nt ex11
Δ3309 nt ex11
Δex11
Δex11
100 bp
100 bp
D
C
IVS16+4 A>G (2)
IVS16+3 G>C
IVS16+5 G>A
C (4)
IVS16+6T>C
IVS16+4 A>G (1)
C (1)
C (5)
NTC
C (3)
C (2)
Ins 65nt
600 bp
ex16
400 bp
F
4794G>A,E1559K
C (1)
NTC
C (5)
C (3)
C (2)
C (4)
ex15
400 bp
Δ11nt ex15
300 bp
E
IVS18+1G>C (1)
IVS18+1G>C (2)
IVS17-2A>G
C (1)
NTC
C (5)
C (3)
C (2)
C (4)
300 bp
ex18
Δex18
200 bp
H
5193G>C,D1692H
C (1)
NTC
C (5)
C (3)
C (2)
C (4)
Ins 153nt
400 bp
ex17
Δex17
200 bp
G
IVS21+13G>T
IVS20-1G>A
IVS20-14C>G
C (1)
NTC
C (5)
C (3)
C (2)
C (4)
200 bp
ex21
Δ8nt ex21
100 bp
Δex21
J
IVS19-1G>T
IVS20+15C>T
C (1)
NTC
C (4)
C (3)
C (5)
C (2)
200 bp
100 bp
Δex20
ex20
Δ13nt ex20
I
IVS22+3A>T
C (4)
C (1)
C (5)
NTC
C (3)
C (2)
200 bp
ex22
100 bp
Δex22
IVS22+4A>G
L
IVS22+2delT (1)
IVS21-1G>T (1)
IVS22+2delT (2)
C (4)
IVS22+2delT (3)
IVS21-1G>T (2)
C (1)
C (5)
NTC
C (3)
C (2)
200 bp
ex22
*
*
*
100 bp
*
*
Δex22
K
5527G>C,G1803A
C (1)
NTC
C (5)
C (2)
C (3)
C (4)
200 bp
ex23
Δex23
100 bp
M
