# Supplementary material for: Analysis of 30 Putative BRCA1 Splicing Mutations in Hereditary Breast and Ovarian Cancer Families Identifies Exonic Splice Site Mutations That Escape In Silico Prediction
Source: PLoS One. 2012 Dec 11;7(12):e50800. doi: 10.1371/journal.pone.0050800 (PMC3519833; doi:10.1371/journal.pone.0050800)
Supplement: Table S1 — Classification, frequencies and in silico characterization of analyzed variants. A: BRCA1 mutations within invariant splice sites; B: Intronic BRCA1 variants outside invariant splice sites; C: Exonic BRCA1 variants; BIC, EVS and 1000 Genomes entries are as of 02/23/2012. BRCA2006 data of the GC-HBOC are as of 04/10/2012. EVS data refers to variation frequencies in the European/American population (rs numbers are given only when frequency data is available). Valuation of variants by the BIC steering committee is given in brackets, when available (yes = clinically important). The consensus values (CVs) for wildtype and mutant splice sites provided by HSF analysis are shown. For HSF prediction, a ΔCV of 10% or more is considered significant. For MaxEntScan analysis, a cutoff value of 20% has been suggested. Differences considered significant are shown in bold./ = no difference between for wildtype and mutant splice sites according to HSF or MaxEntScan. (DOC) [file pone.0050800.s002.doc]

| **BIC nomenclature** | **BIC entries** | **BRCA2006 entries** | **HGMD classification** | **EVS entries** | **HSF matrices** | | | **MaxEntScan** | | |
| --- | --- | --- | --- | --- | --- | --- | --- | --- | --- | --- |
| **A: *BRCA1* variants within invariant splice sites** | | | | | **wild type** | **mutant** | **%** | **wild type** | **mutant** | **%** |
| IVS2-1G>C | / | 4 | disease causing (CS05565) | / | 77.27 | 48.32 | **-37.46** | 7.05 | -1.01 | **-114.33** |
| IVS4-1G>C | / | 2 | / | / | 77.83 | 48.88 | **-37.19** | 8.19 | 0.13 | **-98.41** |
| IVS5+1G>C | 1 (yes) | 1 | disease causing (CS032400) | / | 78.08 | 51.24 | **-34.37** | 7.84 | -0.42 | **-105.36** |
| IVS17-2A>G | / | / | / | / | 91.29 | 62.35 | **-31.71** | 8.96 | 1.01 | **-88.73** |
| IVS18+1G>C | 2 (yes) | 8 | disease causing (CS982091) | / | 82.40 | 55.57 | **-32.57** | 7.96 | -0.30 | **-103.77** |
| IVS18-2delA | 8 (yes) | / | disease causing (CD003507) | / | 83.79 | 54.84 | **-34.55** | 8.78 | 1.73 | **-80.30** |
| IVS19+1delG | / | 3 | / | / | 98.24 | 21.58 | **-78.04** | 11.08 | -12.23 | **-210.4** |
| IVS19+2T>G | / | / | / | / | 98.24 | 71.40 | **-27.32** | 11.08 | 3.43 | **-69.04** |
| IVS19-1G>T | / | / | / | / | 86.33 | 57.38 | **-33.53** | 9.36 | 0.76 | **-91.88** |
| IVS20-1G>A | 3 (yes) | 2 | disease causing (CS973718) | / | 93.65 | 64.70 | **-30.91** | 13.07 | 4.32 | **-66.95** |
| IVS21-1G>T | / | 1 | disease causing (CS014608) | / | 80.46 | 51.51 | **-35.98** | 8.67 | 0.07 | **-99.19** |
| IVS22+2delT | 1 (yes) | 3 | / | / | 83.72 | 33.89 | **-59.52** | 9.49 | -16.44 | **-273.23** |
| **B: Intronic *BRCA1* variants outside invariant splice sites** | | | | |  |  |  |  |  |  |
| IVS4-18T>G | 2 (unknown) | 2 | / | / | / | / | / | 8.19 | 6.96 | -15.02 |
| IVS5+23T>A | **/** | 1 | / | / | / | / | / | / | / | / |
| IVS9-34T>C | / | / | / | / | / | / | / | / | / | / |
| IVS11+3A>G | 5 (unknown) | 3 | / | / | 74.96 | 73.81 | -1.55 | 5.64 | -1,06 | **-118.79** |
| IVS16+3G>C | 11 (yes) | 21 | disease causing (CS031770) | / | 81.24 | 77.38 | -4.76 | 5.91 | -2,36 | **- 139.93** |
| IVS16+4A>G | / | 1 | / | / | 81.24 | 72.90 | **-10.27** | 5.91 | -1.85 | **-131.30** |
| IVS16+5G>A | / | / | / | / | 81.24 | 69.08 | **-14.97** | 5.91 | -5.74 | **-197.12** |
| IVS18-6C>A | 1 (unknown) | 2 | / | / | 83.79 | 80.37 | -4.09 | 8.78 | 7.37 | -11.96 |
| IVS20+15C>T | / | / | / | / | / | / | / | / | / | / |
| IVS21+13G>T | / | 1 | / | / | / | / | / | / | / | / |
| IVS22+3A>T | / | 1 | / | / | 83.72 | 78.69 | -6.00 | 9.49 | 2.03 | **-78.61** |
| IVS22+4A>G | / | 1 | / | / | 83.72 | 75.38 | -9.96 | 9.49 | 5.67 | **-40.25** |
| **C: Exonic *BRCA1* variants** | | | | |  |  |  |  |  |  |
| 710C>T,C197C | 31 (unknown) | 34 | probable disease causing (CM051394) | 12/7020 (rs1799965) | 98.84 | 96.86 | -2.00 | 10.67 | 8.83 | -17.2 |
| 787A>G,K223R | / | 1 | / | / | 79.94 | 79.13 | -1.01 | 5.98 | 4.85 | -18.9 |
| 4304G>A,Q1395Q | 6 (yes) | 4 | / | / | 85.50 | 74.92 | **-12.40** | 8.59 | 1.89 | **-78.00** |
| 4794G>A,E1559K | 2 (yes) | 3 | / | / | 83.37 | 72.79 | **-12.69** | 6.84 | -1.04 | **-115.20** |
| 5193G>C,D1692H | 3 (yes) | 1 | / | / | 76.86 | 65.84 | **-14.33** | 7.48 | -5.83 | **-177.90** |
| 5527G>C,G1803A | 3 (unknown) | 1 | / | / | 83.39 | 83.15 | -0.29 | 4.86 | 4.29 | -11.73 |

**Table S1**
