# Supplementary material for: Analysis of 30 Putative BRCA1 Splicing Mutations in Hereditary Breast and Ovarian Cancer Families Identifies Exonic Splice Site Mutations That Escape In Silico Prediction
Source: PLoS One. 2012 Dec 11;7(12):e50800. doi: 10.1371/journal.pone.0050800 (PMC3519833; doi:10.1371/journal.pone.0050800)
Supplement: Table S2 — Oligonucleotides used for non-quantitative RT-PCR (A) and quantitative real-time RT-PCR analyses (B). (DOC) [file pone.0050800.s003.doc]

| **amplicon** | **forward Primer** | **reverse Primer** | **amplicon length (wt)** | **comment** |
| --- | --- | --- | --- | --- |
| **A: Oligonucleotides used for non-quantitative RT-PCR** | | | | |
| *BRCA1* exon 2-5 | TAT CTG CTC TTC GCG TTG AAG | GGT TGA GAA GTT TCA GCA TGC | 152 |  |
| *BRCA1* exon 3-6 | GGA GTT GAT CAA GGA ACC TG | ACA AGT TGA CTA AAT CTC GTA C | 164 |  |
| *BRCA1* exon 8-10 | GTG AGA ACT CTG AGG ACA AAG | CAG AAT CCA AAC TGA TTT CAT C | 177 |  |
| *BRCA1* exon 9-11 | GAA GAT ACC GTT AAT AAG GCA AC | GCC ACA TGG CTC CAC ATG | 263 |  |
| *BRCA1* exon 10-12 | GAT GAA ATC AGT TTG GAT TCT G | TGT CAC TCT GAG AGG ATA GC | 3531 |  |
| *BRCA1* exon 14-16 | AGT GAA TAC CCT ATA AGC CAG | GCC AAC ACG AGC TGA CTC | 392 |  |
| *BRCA1* exon 11-13 | GTG AAT TGG AAG ACT TGA CTG | CAC AGC TTC TAG TTC AGC CAT | 350 |  |
| *BRCA1* exon 15-17 | TAG ATG ATA GGT GGT ACA TGC | CTG GCA AAC TTG TAC ACG AG | 498 |  |
| *BRCA1* exon 16-18 | GAG TCA GCT CGT GTT GGC | CGT TCA CAC ACA AAC TCA GC | 340 |  |
| *BRCA1* exon 17-20 | CTC GTG TAC AAG TTT GCC AG | CTG GGA TTC TCT TGC TCG C | 278 |  |
| *BRCA1* exon 18-20 | GCT GAG TTT GTG TGT GAA CG | CTG GGA TTC TCT TGC TCG C | 191 |  |
| *BRCA1* exon 19-21 | GTG ACC CAG TCT ATT AAA GAA AG | TGG TGA AGG GCC CAT AGC | 165 |  |
| *BRCA1* exon 20-22 | GCG AGC AAG AGA ATC CCA G | GCA CCA CAC AGC TGT ACC | 116 |  |
| *BRCA1* exon 21-23 | GCT ATG GGC CCT TCA CCA | GTC CAG GCA TCT GGC TG | 144 |  |
| *BRCA1* exon 22-24 | GGT ACA GCT GTG TGG TGC | AGT GCT ACA CTG TCC AAC AC | 182 |  |
| **B: Oligonucleotides used for quantitative real-time RT-PCR** | | | | |
| *BRCA1* exon 6/7 | GAC ACA GGT TTG GAG TAT GC | TCG GGT TCA CTC TGT AGA AG | 144 | forward primer spans exon 6/7 boundary |
| *BRCA1* exon 2/3/5/6 | GTG TCC CAT CTG TCT GGA GT | CTT GTA GGC TCC TTT TGG TTA TAT C | 153 | forward primer spans exon 2/3 boundary  reverse primer spans exon 5/6 boundary |
| *BRCA1* exon 2/3/6 | ATC TGC TCT TCG CGT TGA AG | CTT GTA GGC TCT TGC AAA ATA TGT | 136 | reverse primer spans exon 3/6 boundary |
| *BRCA1* exon 8/9/10 | CCTTGGAACTGTGAGAACTCT | CTCCCACACTGCAATAAGTTG | 128 | reverse primer spans exon 9/10 boundary |
| *BRCA1* exon 8/10 | CCTTGGAACTGTGAGAACTCT | GATCTCCCACACCAATTCAATG | 84 | reverse primer spans exon 8/10 boundary |
| *BRCA1* exon 8/11 | CCTTGGAACTGTGAGAACTCT | CACAAGCAGCCAATTCAATG | 83 | reverse primer spans exon 8/11 boundary |

**Table S2**
